# Influence of oral mucosal lesions and oral symptoms on oral health related quality of life in dermatological patients: a cross sectional study in Sudan

**DOI:** 10.1186/1472-6831-12-19

**Published:** 2012-07-08

**Authors:** Nada M Suliman, Anne C Johannessen, Raouf W Ali, Hussein Salman, Anne N Åstrøm

**Affiliations:** 1 Section for pathology, The Gade Institute, University of Bergen, Bergen, Norway; 2 Haukeland University Hospital, Bergen, Norway; 3 Faculty of Dentistry, University of Science and Technology, Umdurman, Sudan; 4 Dermatology Clinic, Khartoum Teaching Hospital, Khartoum, Sudan; 5 Department of Clinical Dentistry, University of Bergen, Bergen, Norway

**Keywords:** dermatology, oral mucosal lesions, oral impact on daily performance, quality of life

## Abstract

**Background:**

There are only few studies considering the impact of oral mucosal lesions (OML) on the oral quality of life of patients with different dermatological conditions. This study aimed to assess the relationship between oral health-related quality of life (OHRQoL) and OML and reported oral symptoms, perceived general and oral health condition and caries experience in adult skin diseased patients attending an outpatient dermatologic clinic in Sudan.

**Methods:**

A cross-sectional survey was carried out with 544 diagnosed skin diseased patients (mean age 37.1 years, 50 % females), during the period October 2008 to January 2009. The patients were orally examined and OML and caries experience was recorded. The patients were interviewed using the Sudanese Arabic version of the OIDP. OHRQoL was evaluated by socio-demographic and clinical correlates according to number of types of OML diagnosed (no OML, one type of OML, > one type of OML) and number and types of oral symptoms.

**Results:**

An oral impact (OIDP > 0) was reported by 190 patients (35.6 %) (mean OIDP total score 11.6, sd = 6.7). The prevalence of any oral impact was 30.5 %, 36.7 % and 44.1 %, in patients with no OML, one type of OML and more than one type of OML, respectively. Number of types of OML and number and types of oral symptoms were consistently associated with the OIDP scores. Patients who reported bad oral health, patients with ≥ 1 dental attendance, patients with > 1 type of OML, and patients with ≥ 1 type of oral symptoms were more likely than their counterparts in the opposite groups to report any OIDP. The odds ratios (OR) were respectively; 2.9 (95 % CI 1.9-4.5), 2.3 (95 % CI 1.5-3.5), 1.8 (95 % CI 1.1-3.2) and 6.7 (95 % CI 2.6-17.5). Vesiculobullous and ulcerative lesions of OML disease groups associated statistically significantly with OIDP.

**Conclusion:**

OIDP was more frequently affected among skin diseased patients with than without OML. The frequency of the impacts differed according to the number of type of OML, oral symptoms, and OML disease groups. Dentists and dermatologists should pay special attention to skin diseased patients because they are likely to experience oral impacts on daily performances.

## Background

Oral mucosal lesions (OML) may be the initial feature or the only clinical sign of mucocutaneous diseases, a group of mainly chronic diseases, commonly observed in a dermatologic practice [1-4]. In a previous study considering Sudanese adults with mucocutaneous diseases attending an outpatient dermatology clinic in Khartoum, the prevalence of patients with OML was high, amounting to 57.9 % [5]. Patients with OML experience a wide range of chronic and recurrent conditions that may have detrimental effect on functioning, social life and psychological well-being.

Evidently, mucocutaneous diseases have impacts on the quality of life of patients comparable to that of other medical conditions [6,7]. Patient reported outcomes in terms of oral health related quality of life (OHRQoL) measures have seldom been assessed in relation to mucocutaneous conditions [8-15]. Whereas the relationship of dental and periodontal status with OHRQoL measures has been examined across various socio-cultural contexts, few studies have considered the impact on OHRQoL of patients with disorders that are of relevance to oral medicine and dermatological practice [11,13,16,17]. This is so, although patient reported outcomes of OHRQoL may provide valuable information, for example by identifying treatment needs, selecting therapies, evaluating treatment outcomes and monitoring patient progress [18].

Several generic and disease specific OHRQoL measures have been developed to provide better understanding of the consequences of oral diseases upon quality of life and to complement traditional clinical measures [6,19]. Whereas specific OHRQoL measures assess impacts that are attributable to specific oral diseases, the generic ones take into account numerous oral conditions, some occurring simultaneously, thus providing information on the wider implications of oral status [20]. One promising generic OHRQoL measure is the Oral Impacts on Daily Performance (OIDP) scale [21,22]. The OIDP was developed to measure oral impacts that seriously affect a person’s daily life. It is based on the conceptual framework of the World Health Organisation’s International Classification of Impairments, Disabilities and Handicaps (ICIDH) [23], which has been amended for dentistry by Locker [24]. The OIDP concentrates only on the measurement of “ultimate” oral impacts, thus covering the fields of disability and handicap [22] . This inventory assesses the impact of oral conditions on basic activities and behaviours that cover the physical, psychological, and social dimensions of daily living. Considering respondent burden, the OIDP is suitable for use in population surveys and clinical practices, not only in terms of being easier when measuring behaviours rather than feeling states, but also in being short. It is originally calculated by multiplying frequency and severity scores of daily performances, providing an overall score for each OIDP item. However, applications of the weighted OIDP scores revealed no significant improvement over the use of OIDP frequency or severity scores [22]. Thus, it has been proposed to use either the frequency or the severity OIDP scores for simplicity and efficiency. Since its development, the OIDP has shown to be reliable and valid in general population based studies [25-28], as well as in studies of patients with specific oral disorders, such as traumatic injuries, periodontal disease and malocclusion [16,17,29]. Although an Arabic version of the 8 item OIDP inventory has been applied previously with Sudanese children [30] and dental attendees from a Sudanese adult population [31], this study necessitated reestablishment of its psychometrical properties. The generic OIDP inventory has yet to be applied in the context of patients with mucocutaneous diseases.

This study aimed to assess the relationship between oral health related quality of life (OHRQoL) and OML and reported oral symptoms, perceived general and oral health condition and caries experience in Sudanese adult skin diseased patients attending an outpatient dermatologic clinic in Sudan.

## Methods

### Sampling procedure

The present study is a part of a cross sectional hospital based study that was carried out from October 2008 to January 2009 [5]. The study was focusing on patients aged 18 years and above with mucocutaneous diseases, attending an outpatient dermatologic clinic at Khartoum Teaching Hospital (KTH). KTH is the largest national hospital in Sudan, located in Khartoum, the capital city. It is an open public and referral hospital receiving patients from all states of the country. A minimum sample size of 500 patients was calculated to estimate differences in OHRQoL between patients with and without oral mucosal lesions assuming the proportions of oral impacts to be 0.60 and 0.40 among patients with and without OML lesions, significance level (two sided test) of 5 % and statistical power of 80 %. All patients (n = 4235) attending the outpatient facility during the survey period were invited to participate in the study. A total of 1540 subjects (36.4 %) initially accepted to participate. Fear of taking biopsy for asymptomatic lesions and time consuming examinations (oral examination, interview, and biopsy when needed) were the main reasons for not volunteering to participate. Of those who initially accepted to participate, 544 (544/1540, 35.3 %) patients were included in the study. Unexplained disappearance of patients and limited financial resources were the main reasons for withdrawal from the study. Thus, the final participation rate was 544/4235, 12.8 %. Confidentiality of the patients was maintained, participants were informed about their oral conditions, and health education was provided. Those who needed dental services were referred to the University of Science and Technology (UST), Faculty of Dentistry, for further investigation and management. Written informed consent or finger print (illiterates) for participation and publication of the study was obtained from patients or their parents/guardians. The research conformed to the Helsinki Declaration, and ethical clearance and approval letters were obtained by the participating institutions’ committees in Sudan (UST and KTH, Department of Dermatology). In Norway, the ethical approval was obtained from the Regional Committee for Medical Research Ethics of Western Norway.

### Survey instrument

A pilot study revealed a considerable number of illiterate patients, and thus a structured questionnaire was interviewer administered by two trained dentists. The interview schedule contained questions regarding socio-demographics, health and oral health related characteristics and lifestyles. The interview schedule was constructed in English and then translated and used in Arabic. Forward and backward translations were performed by two independent Sudanese professional translators in Arabic and English language. Sensitivity to culture and selection of appropriate words were considered by use of simple common Arabic words.

*OHRQoL* was assessed using the eight items OIDP frequency inventory [21,22]; ‘During the past 6 months, how often have problems with your mouth and teeth caused you any difficulty with: eating and chewing food; speaking and pronouncing clearly; cleaning teeth; sleeping and relaxing; smiling and showing teeth without embarrassment; maintaining usual emotional state; carrying out major work and social role, and enjoying contact with people?’. Each item was assessed using a 5-point scale: (1) Never affected; (2) Less than once a month; (3) Once or twice a month; (4) Once or twice a week; (5) Every, or nearly every day. Initially, an additive sum score (OIDP ADD) was constructed from the 8 items as originally scored (1–5, range 8–40). Secondly, each OIDP frequency item was dichotomised, yielding the categories: (0) never affected (including the original category 1), (1) affected (including the original categories 2, 3, 4, and 5). Simple count scores (SC) were created for the OIDP by adding the eight dichotomised variables. For the purpose of cross-tabulation and logistic regression analysis, the OIDP SC scores (0–8) were dichotomised as 0 = no daily performance affected and 1 = at least one daily performance affected. The distribution of the OIDP SC scores supported this cut-off point.

Socio-demographic characteristics were assessed in term of gender, age, education, tribes, marital status, and place of residence. Gender was assessed as: (1) female; (2) male. Age was recorded by asking, ‘how old are you?’ and the answers were dichotomized into 2 equally sized groups; (0) 18–32 years and (1) 33 + years. Participants were classified according to their educational level using five categories: (1**)** illiterate; (2) primary school; (3) secondary school; (4) university; (5) higher studies. Two dummy variables were constructed yielding the categories 0 = lower education (including the original categories 1 and 2) and 1 = higher education (including the original categories 3, 4, and 5). Medical condition was assessed as a sum score of the following: heart diseases, hypertension, asthma, diabetes, liver diseases, hepatitis /jaundice, anaemia, bleeding disorders, kidney diseases, rheumatoid arthritis, allergy, cancer, epilepsy, stomach ulcer, intestinal disorders, psychiatric/mental disorders, respiratory disorders, and pregnancy. The sum scores were dichotomized into 0 = none and 1 = ≥ one. Perceived health status was recorded from (1) very bad to (4) very good. Two dummy variables were created in terms of 0 = good and 1 = bad. *Perceived oral health status* was measured using a 5-point rating by asking; ‘How do you consider the present condition of your mouth and teeth?’ with response categories: (1) very bad; (2) bad; (3) neither good nor bad; (4) good; (5) very good. This variable was dichotomized in terms of 0 = good (including the original categories 4 and 5) and (1) = bad (including the original categories 1, 2 and 3). Reported oral symptoms were assessed by the question ‘During the previous 6 months have you experienced: dental pain/toothache, abscessed tooth, dry mouth, bleeding gums, infected sore gums, tooth decay, or broken tooth. Each symptom was assessed as present (1) and absent (0)*.* Frequency of dental attendance was assessed by asking ‘How many times have you attended a dentist during the previous 2 years?’ with response categories: (1) once; (2) twice, (3) more than twice; (4) never. A dummy bivariable was constructed yielding the response categories 1 = attended dental clinic (including the original categories 1, 2 and 3) and 0 = never attended dental clinic.

### Clinical examination

Systematic comprehensive extra-oral and intra-oral clinical examinations based on visual inspection and palpation, following the World Health Organization (WHO) criteria for field surveys [32], were carried out by a dentist (NMS) who received a standard training in diagnosis of OML before the data collection (The Gade Institute, Section for Pathology, and Department of Clinical Dentistry, Section for Oral Surgery and Oral Medicine, University of Bergen, Norway). Caries experience was assessed under field conditions and scored according to the criteria described by the WHO [33]. A tooth was recorded as decayed when a cavity was apparent on visual inspection. Missing tooth was recorded if there was a history of extraction because of pain and/ or a cavity prior to extraction. DMFT, was computed as the sum of decayed, missing and filled teeth and dichotomized into caries free DMFT = 0 and having any caries experience DMFT > 0. The oral clinical examination and information with respect to OML and oral habits have been detailed elsewhere [5].

### Diagnostic criteria for oral mucosal lesions

An OML was defined as any abnormal change or any swelling on the oral mucosal surface. A single lesion with confirmed diagnosis was referred to as a ‘type of OML’. Diagnostic criteria for OML were based on Axéll criteria and those defined in earlier studies and reviews [32,34,35].

### Statistical analysis

Data were analysed using PASW Statistics version 18.0 (SPSS Inc., Chicago). Non-parametric statistics were used because the OIDP-total scores were not normally distributed. Bivariate relationships were assessed using cross-tabulation, chi-square statistics and Mann Whitney - U test. Internal consistency reliability was assessed using Cronbach’s alpha. To adjust for potential confounding factors, multiple variable logistic regression analyses were performed and OR and Nagelkerkes R^2^ were calculated. The relationship between OIDP and number of different types of OML was assessed in unadjusted and fully adjusted models. The relationship between OIDP and each type of reported symptoms and OML disease groups was assessed in unadjusted, fully adjusted and mutually adjusted models.

## Result

### Sample profile

A total of 544 patients with mucocutaneous diseases participated in the present study. The mean age was 37.1 years, sd = 15.9 years (range 18–85), 50 % were females, 77 % were permanent residents of Khartoum during the previous 5 years, 47.8 % belonged to the older age group (33–85 years) and 50.1 % reported higher education. A total of 57.9 % of the patients were diagnosed with at least one clinically recognized type of OML. Full details of the prevalence of OML (types and group diseases) of the participants studied are described elsewhere [5]. A particular type of OML was recorded only once although it could be manifested at several locations in the same patient. The age of patients affected by OML ranged from 18 to 81 years, with an average of 38.6 years (sd = 16.5). A total of 6 OML group diseases, each including at least 20 patients, were recognized for the present study. *Tongue lesions* were the most frequently diagnosed OML group diseases (23.3 %) followed in descending order by *white lesions* (19.1 %), *red and blue lesions* (11 %), *vesiculobullous diseases* (6 %), *oral ulcerative lesions* (4.5 %) and *pigmented lesions* (3.9 %). Table 1 depicts the distribution of patients’ socio-demographic, behavioural, oral symptoms and clinical features by number of types of OML. As shown, a total of 89.9 % had caries experience, 84.4 % reported more than one oral symptom and 45.5 % reported more than one systemic health condition. The most and least frequently reported conditions were tooth decay (57.1 %) and abscess (9.6 %), respectively.

**Table 1:** Socio-demographics, behavioural- and clinical characteristics of patients with mucocutaneous diseases according to number of types of OML

	**No OML% (n)**	**One type of OML% (n)**	**> One type of OML% (n)**	**Total% (n)**
Gender				
Female	56.3 (129)	49.5 (100)	38.1 (43)	50.0 (272)
Male	43.7 (100)	50.5 (102)	61.9 (70)	50.0 (272)
Age				
Younger (18–32 years)	57.9 (132)	53.3 (105)	37.8 (42)	52.2 (279)
Older (33–85 years)	42.2 (96)	46.2 (90)	62.2 (69)	47.8 (255)
Education				
Low	46.3 (105)	49.5 (97)	58.0 (65)	49.9 (267)
High	53.7 (122)	50.5 (99)	42.0 (47)	50.1 (268)
Systemic condition				
None	62.4 (143)	50.0 (101)	46.9 (53)	54.6 (297)
≥ one	37.6 (86)	50.0 (101)	53.1 (60)	45.4 (247)
Perceived general health status				
Bad	31.1 (71)	27.1 (54)	44.1 (49)	32.3 (174)
Good	68.9 (157)	72.9 (145)	55.9 (62)	67.7 (364)
Perceived oral health status				
Bad	36.0 (82)	39.5 (79)	36.6 (41)	37.4 (202)
Good	64.0 (146)	60.5 (121)	63.4 (71)	62.6 (338)
Dental attendance				
Never attended dental clinic	67.3 (152)	60.0 (120)	52.2 (59)	61.6 (331)
Attended dental clinic	32.7 (74)	39.4 (78)	47.8 (54)	38.4 (206)
Number of reported oral symptoms				
None	18.2 (38)	15.0 (26)	11.2 (11)	15.6 (75)
≥ one	81.8 (171)	85.0 (147)	88.8 (87)	84.4 (405)
Specific reported oral symptoms				
Dental pain (yes)	44.6 (100)	45.2 (89)	48.6 (54)	45.7 (243)
Tooth decay (yes)	58.7 (132)	50.8 (101)	65.5 (72)	57.1 (305)
Abscess (yes)	9.0 (20)	9.6 (19)	10.7 (12)	9.6 (51)
Broken tooth (yes)	14.4 (32)	19.1 (36)	11.4 (12)	15.5 (80)
Dry mouth (yes)	21.3 (48)	22.1 (43)	22.0 (24)	21.7 (115)
Bleeding gum (yes)	36.4 (83)	37.9 (75)	44.1 (49)	38.5 (207)
Infected sore gum (yes)	12.4 (28)	16.7 (32)	23.4 (26)	16.3 (86)
OML pain (yes)	1.3 (3)	19.3 (39)	11.5 (13)	10.1 (55)
DMFT				
DMFT = 0	8.7 (20)	12.9 (26)	8.0 (9)	10.1 (55)
DMFT > 0	91.3 (209)	87.1 (176)	92.0 (104)	89.9 (489)

The total number in the different categories did not add to 544 owing to missing values.

#### Psychometric properties of the OIDP

In the present study, small number of missing responses (2–11) adds support to face validity of the OIDP frequency inventory. As depicted in Table 2, One hundred and ninety patients (35.6 %) perceived at least one oral impact (OIDP > 0). The mean OIDP ADD was 11.6 (sd = 6.7) The prevalence of any oral impact was 30.5 %, 36.7 % and 44.1 % in patients with respectively, no OML, one type of OML and more than one type of OML. A problem with *eating* was the most frequently reported impact. Problems with *workcontact people*, and *smiling* were the least frequently reported impacts across the three OML groups as well as in the total study group. As shown in Table 3, Cronbach’s alpha for the OIDP in the study group was 0.89 with corrected item-total correlation ranging from 0.57 (*smiling*) to 0.70 (*emotional state*)***.*** The standardized items alpha in the separate groups was 0.81 (no OML), 0.89 (one type of OML) and 0.92 (> one type of OML). The corrected item-total correlation across the three groups was above the minimum level of 0.2 required for including an item into a scale [36].

**Table 2:** Percentage distribution and mean scores (SD) for the eight OIDP frequency items and the OIDP ADD score in skin diseased patients by number of types of OML

**OIDP items**	**No OML N = 229**		**One type of OML N = 202**		**> One type of OML N = 113**		**Total population N = 544**	
	**Affected% (n)**	**Mean (SD)**	**Affected% (n)**	**Mean (SD)**	**Affected% (n)**	**Mean (SD)**	**Affected% (n)**	**Mean (SD)**
Eating	26.4 (60)	1.7 (1.4)	33.0 (66)	2.0 (1.6)	39.8 (45)	2.2 (1.6)	31.7 (171)	1.9 (1.5)
Emotional state	16.2 (37)	1.5 (1.2)	25.9 (51)	1.8 (1.4)	30.4 (34)	1.9 (1.5)	22.7 (122)	1.7 (1.3)
Cleaning	17.1 (39)	1.5 (1.2)	23.4 (46)	1.7 (1.4)	26.8 (30)	1.8 (1.5)	21.4 (115)	1.6 (1.3)
Sleeping	12.7 (29)	1.3 (1.0)	18.3 (36)	1.5 (1.2)	16.2 (18)	1.5 (1.2)	15.5 (83)	1.4 (1.1)
Speaking	4.80 (11)	1.1 (0.6)	8.0 (16)	1.2 (0.8)	15.9 (18)	1.4 (1.1)	8.3 (45)	1.2 (0.8)
Contact people	4.40 (10)	1.1 (0.6)	9.5 (19)	1.3 (1.0)	10.6 (12)	1.3 (1.0)	7.6 (41)	1.2 (0.9)
Major work	4.40 (10)	1.1 (0.6)	7.7 (15)	1.2 (0.9)	9.8 (11)	1.3 (1.0)	6.7 (36)	1.2 (0.8)
Smiling	1.8 (4)	1.0 (0.4)	8.5 (17)	1.2 (0.8)	11.5 (13)	1.4 (1.2)	6.3 (34)	1.2 (0.8)
OIDP > 0	30.5 (69)	0.8 (1.5)	36.7 (72)	1.3 (2.1)	44.1 (49)	1.5 (2.3)	35.6 (190)	1.1 (1.9)
OIDP ADD		10.4 (4.8)		12.1 (7.2)		13.1 (8.4)		11.6 (6.7)

Means and % varied according to the total number of respondents in each OIDP item due to lack of information in 2–11 patients across the OIDP items.

**Table 3:** Corrected item total correlation and Cronbach’s alpha of OIDP by number of types of OML

**No OML (N = 226) Cronbach's Alpha (Standardized Items) = 0.811**		
**OIDP items**	**Corrected Item-Total Correlation**	**Cronbach's Alpha if Item Deleted**
Eating	.675	.757
Speaking	.447	.792
Cleaning	.592	.767
Smiling	.269	.808
Sleeping	.634	.759
Emotional state	.655	.755
Carrying out major work	.564	.779
Contact	.391	.796
One type of OML (N = 196)		
Cronbach's Alpha (Standardized Items) = 0.894		
Eating	.721	.867
Speaking	.586	.878
Cleaning	.713	.865
Smiling	.614	.876
Sleeping	.715	.865
Emotional state	.680	.870
Carrying out major work	.646	.874
Contact	.683	.870
> One type of OML (N = 111)		
Cronbach's Alpha (Standardized Items) = 0.921		
Eating	.700	.909
Speaking	.711	.906
Cleaning	.751	.902
Smiling	.657	.910
Sleeping	.751	.902
Emotional state	.779	.900
Carrying out major work	.766	.903
Contact	.747	.904
Total study population (N = 533)		
Cronbach's Alpha (Standardized Items) = 0.890		
Eating	.694	.863
Speaking	.606	.869
Cleaning	.692	.859
Smiling	.572	.872
Sleeping	.698	.858
Emotional state	.703	.858
Carrying out major work	.671	.864
Contact	.642	.866

The association between the frequency of oral impacts (OIDP total >0) and factors known to be associated with oral health; socio-demographic-, clinical and behavioural variables were assessed using cross tabulation and multiple variable logistic regression analyses. As depicted in Table 4, the frequency of subjects having at least one impact (OIDP > 0) increased significantly with increasing number of types of OML both in unadjusted and adjusted analysis with subjects having more than one type of OML being about twice as likely as their counterparts without OML to report oral impacts (OR 1.8 (95 % CI 1.1-3.2). Perceived oral health status remained statistically significantly associated with OIDP after having included all variables in the model. Multiple logistic regression analysis revealed that socio-demographic, behavioural variables and medical conditions entered in the first step explained 20 % of the variance (Nagelkerke R^2^ = 0.20). Entering number of types of OML in step 2 raised the explainable variance by 1 % (Nagelkerke R^2^ = 0.21).

**Table 4:** Unadjusted and adjusted associations of OIDP with socio-demographics, behaviours and number of types of OML in skin diseased patients (n = 544). Percentage (n), odds ratio (OR) and 95 % Confidence Interval (CI)

**Variables**	**OIDP > 0N = 190% (n)**	**Unadjusted OR (95 % CI)**	**Adjusted OR (95 % CI)**
Gender			
Male	30.7 (81)	1	1
Female	40.5 (109)*	1.5 (1.0-2.2)	1.4 (0.9-2.1)
Age			
18-32 yr	34.7 (95)	1	1
33-85 yr	36.4 (91)	1.0 (0.7-1.5)	0.9 (0.6-1.5)
Education			
Lower	31.2 (82)	1	1
Higher	39.3 (103)*	1.4 (0.9-2.0)	1.4 (0.9-2.3)
Perceived oral health status			
Good	24.5 (82)	1	1
Bad	54.8 (108)**	3.7 (2.5-5.4)	2.9 (1.9-4.5)**
Perceived health status			
Good	30.5 (110)	1	1
Bad	46.7 (79) **	2.0 (1.3-2.9)	1.5 (0.99-2.4)¶
Dental attendance			
Never attended dental clinic	27.4 (90)	1	1
Attended dental clinic	48.8 (98)**	2.5 (1.7-3.6)	2.3 (1.5-3.5)**
Medical conditions			
None	30.1 (87)	1	1
At least one	42.2 (103)*	1.6 (1.1-2.4)	1.3 (0.8-2.1)
Number of types of OML ♯			
None	30.5 (69)		1
One	36.7 (72)		1.2 (0.7-1.9)
>one	44.1 (49)*		1.8 (1.1-3.2)*

* p < 0.05, ** p < 0.001, ¶ p = 0.05)

***♯*** Number of types of OML; no OML (p = 0.06), one type OML (p = 0.3), >one type OML (p = 0.02)

The sum of the categories listed may not equal the total number due to lack of information.

As shown in Table 5, six out of eight specific symptoms were associated with impaired OHRQoL in adjusted logistic regression analyses. When all reported symptoms were accounted for, only OML pain (OR 10.3, 95 % CI 4.2-25.4), infected sore gums (OR 4.1, 95 % CI 1.9-8.6), dental pain (OR 3.1, 95 % CI 1.8-5.3) and tooth decay (OR 1.8, 95 % CI 1.0-3.1) remained statistically significantly associated with OIDP. Pain associated with mucosal lesion had the strongest impact OR 10.2 (95 % CI 4.2-25.0), and dry mouth the weakest impact OR 0.9 (95 % CI 0.4-1.7) on OIDP. As depicted in Table 6, the OML disease groups of vesiculobullous and ulcerative lesions discriminated statistically significantly between subjects with and without OIDP in adjusted as well as in mutually adjusted logistic regression analyses. A total of 72.4 % versus 33.5 % (p < 0.001) of the participants with and without vesiculobullous lesions and 77.3 % versus 33.9 % of participants with and without oral ulcerative lesions (p < 0.001) had oral impacts on their daily performances. When adjusting for socio-demographics, subjects with vesiculobullous lesions were 7.4 times OR 7.4 (95 % CI 2.9-18.8) and subjects with oral ulcerative lesions were 5.7 times OR 5.7 (95 % CI 1.9-16.9) more likely than their counterparts without those OML disease groups to report oral impacts. The corresponding mutually adjusted ORs were 8.2 (95 % CI 3.2-20.9) and 6.7 (95 % CI 2.2-20.0).

**Table 5:** Unadjusted, adjusted and mutually adjusted associations of OIDP with reported oral symptoms in skin diseased patients (n = 544). Percentages (n), odds ratios (OR) and 95 % confidence interval (CI)

**Reported symptoms previous 6 months**	**OIDP > 0% (n)**	**Unadjusted OR (95 % CI)**	**Adjusted**^**a**^**OR (95 % CI)**	**Mutually adjusted**^**b**^**OR (95 % CI)**
Dental pain				
No	20.0 (57)	1	1	1
Yes	55.2 (132)**	4.9 (3.3-7.2)	4.3 (2.8-6.6)**	3.1 (1.8-5.3)**
Tooth decay				
No	21.8 (49)	1	1	1
Yes	46.4 (140)**	3.1 (2.1-4.5)	2.8 (1.8-4.3)**	1.8 (1.0-3.1)*
Abscess				
No	31.7 (150)	1	1	1
Yes	74.0 (37)**	6.1 (3.1-11.8)	4.7 (2.3-9.5)**	2.3 (1.0-5.3)*
Broken tooth				
No	36.2 (155)	1	1	
Yes	35.4 (28)	0.9 (0.5-1.5)	0.8 (0.4-1.4)	- -
Dry mouth				
No	32.7 (133)	1	1	1
Yes	46.5 (53)*	1.7 (1.1-2.7)	1.5 (0.9-2.4)	0.9 (0.4-1.7)
Bleeding gum				
No	27.0 (88)	1	1	1
Yes	49.3 (100)**	2.6 (1.8-3.7)	2.5 (1.6-3.7)**	1.3 (0.7-2.2)
Infected sore gum				
No	27.8 (122)	1	1	1
Yes	78.0 (64)**	9.2 (5.2-16.2)	7.4 (4.0-13.4)**	4.1 (1.9-8.6)**
OML pain				
No	31.3 (151)	1	1	1
Yes	76.5 (39)**	7.1 (3.6-13.9)	11.2 (5.2-23.9)**	10.3 (4.2-25.4)**
Number of symptoms				
None	9.6 (7)	1	1	
At least one	41.0 (164)**	6.5 (2.9-14.6)	6.7 (2.6-17.5)**	

*p < 0.05, **p < 0.001

a) Adjusted for sex, age, education, perceived health status, dental attendance and medical condition.

b) Adjusted for sex, age, education, perceived health status, dental attendance, medical condition and other symptoms.

The sum of the categories listed may not equal the total number due to lack of information.

**Table 6:** Models for the association between OML disease groups and OIDP (n = 544)

**OML disease groups**	**N**	**OIDP > 0N = 190% (n)**	**Unadjusted OR (95 % CI)**	**Adjusted**^**a**^**OR (95 % CI)**	**Mutually adjusted**^**b**^**OR (95 % CI)**
Tongue lesions					
No	417	34.8 (142)	1	1	
Yes	127	38.4 (48)	1.1 (0.7-1.7)	1.0 (0.6-1.6)	
White lesions					
No	440	34.7 (149)	1	1	
Yes	104	39.8 (41)	1.2 (0.8-1.9)	1.3 (0.7-2.1)	
Red and blue lesions					
No	484	36.1 (171)	1	1	
Yes	60	32.3 (19)	0.8 (0.4-1.4)	0.9 (0.9-1.0)	
Vesiculobullous lesions					
No	513	33.5 (169)	1	1	1
Yes	31	72.4 (21)**	5.2 (2.2-11.9)	7.4 (2.9-18.8)**	8.2 (3.2-20.9)**
Oral ulcerative lesions					
No	520	33.9 (173)	1	1	1
Yes	24	77.3 (17)**	6.6 (2.4-18.3)	5.7 (1.9-16.9)*	6.7 (2.2-20.0)*
Pigmented lesions					
No	523	35.7 (183)	1	1	
Yes	21	35.0 (7)	0.9 (0.3-2.4)	0.7 (0.2-2.3)	

*p < 0.05, **p < 0.001

a) Adjusted for sex, age, education, perceived health status, dental attendance and medical condition.

b) Adjusted for sex, age, education, perceived health status, dental attendance, medical condition and other OML disease groups.

The sum of the categories listed may not equal the total number due to lack of information.

## Discussion

This is the first study considering OHRQoL in patients with various mucocutaneous diseases, using an Arabic version of the OIDP frequency inventory. Arabic versions of OHRQoL instruments such as the Oral Health Impact Profile (OHIP-14), the Geriatric Oral Health Assessment Index (GOHAI) and the OHQoL-UK inventory have been reported to be reliable and valid for use in adult populations from Saudi Arabia, Egypt and Syria [37-39]. The results of this study indicate that, when used with patients having mucocutaneous diseases, the Arabic OIDP version is valid and reliable demonstrating psychometric properties similar to the original English version [27] as well as the Thai [26], Greek [28] and Norwegian versions of the OIDP [40]. Moreover, the OIDP has shown to be usable across various subgroups of the Sudanese population [30,31], first applied as a self-administered questionnaire in dental attendees from the general population, secondly in personal interviews with schoolchildren and more recently in personal interviews with patients in a dermatologic clinic. Thus, internal consistency reliability in terms of Cronbach’s alphas of 0.89 was satisfactory and well above the recommended level of 0.70 [36]. Moreover, the corrected item-total correlation coefficients were above the minimum level of 0.20 for inclusion of an item into a scale across patients with and without OML [36]. Although no approach guarantees cross-cultural equivalence, the Arabic version of OIDP seemed to preserve the overall concepts of the English version and did not differ in terms of sequence of questions, the Likert scale and the recall memory period (6 months) used. Notably, the respondents had few difficulties in completing the 8 item OIDP interview. This highlights the feasibility of employing the Arabic version of the OIDP frequency inventory in oral medicine and dermatologic clinical settings in Sudan. Recognizing the frequency and severity of the OIDP scores to have similar predictive power, using the OIDP frequency score in this study, should be the better single choice because of its better reproducibility [22]. However, the degree of impact could not be accounted for by this model.

According to the present results, the frequency of oral impacts varied systematically and in the expected direction with self-reported oral health status, clinical dentition status and number of reported oral symptoms across patients having none, at least one and more than one type of OML. Moreover, patients having more than one type of OML were more likely to report oral impacts than their counterparts without OML and with only one type of OML, suggesting a cause – effect relationship. Notably, cross-sectional studies cannot provide definite information about cause - and- effect relationships since both predictor and outcome variables have been measured at the same point in time. Longitudinal studies are needed to improve the interpretation of factors influencing OIDP in adult patients with OML. The moderate fit of the overall multivariable model indicates that other essential variables were not included in the model. Types of OML have fluctuated from asymptomatic lesions (snuff dipper lesions) to the most chronic and painful one (oral pemphigus vulgaris) [5]. In the future, stratified analysis of types of OML should be considered as chronic OML has proven to decrease quality of life [41].

Both type and number of reported oral symptoms discriminated between patients with and without oral impacts (OIDP > 0). Dental attendance was one of the strongest predictors of oral impact in this study.The association between dental attendance and improved oral health has been widely documented [42]. However, in this study, dental attendance was associated with deteriorated OHRQoL.That pattern might reflect perceived treatment need among the study population [43]. This is consistent with results reported previously [44,45] . Although pain was the second less commonly reported symptom, it emerged as the strongest predictor of oral impacts among the symptoms investigated both in adjusted and mutually adjusted logistic regression analyses. This is consistent with the multidimensional nature of pain that affect physical, social and psychological well-being [10,46]. In the context of oral health, oral pain influences eating, drinking, and other oral every day activities. Conversely, the highly prevalent condition of tooth decay had a small negative impact on OIDP. This might be attributed to the fact that patients learn to cope with commonly occurring symptoms and conditions that become less disabling with recurrence.

The present results corroborate findings with other OHRQoL measures. Generic OHRQoL measures (OHRQoL-UK measure and OHIP-14 were proven to be valid and reliable in patients with oral lichen planus (OLP) [10]. Moreover, oral health in patients with symptomatic OLP was reported to have an increased burden on their life quality compared to those with non-symptomatic OLP. Mc Grath et al [8] found that patients with ulcers, erosions and symptomatic oral lesions had bad OHIP-14 scores, suggesting that they had increased quality of life impairments compared to their counterparts with non-symptomatic lesions. Similar results have been presented in studies of patients with Behçet’s disease using the OHIP-14 inventory [47]. In another study of UK patients, attending an outpatient oral medicine clinic, Llewellyn et al [9] found that patients with stomatological disease to have higher levels of functional limitations, physical pain and psychological discomfort than the general population. Oral ulceration associated with Behçet's disease and recurrent aphthous stomatitis (RAS) have been reported to impair life satisfaction and the performance of daily activities [11,13]. A Spanish study comparing OHRQoL in patients with OLP with healthy controls concluded that impairments were greatest in the former group of patients across all dimensions of the OHIP inventory [14]. According to this study results, about 30-40 % of the patients with the OML disease groups of tongue lesions, white lesions, red and blue lesions and pigmented lesions reported oral impacts. On the other hand, the impact frequency among patients suffering oral ulcerative conditions and vesiculobullous diseases amounted to 77 % and 72 %, respectively. A previous study revealed that RAS and pemphigus vulgaris were the most frequently occurring diagnosis among oral ulcerative conditions and vesiculobullous diseases in mucocutaneous diseased patients attending the KTH [5] . Evidence that RAS has the highest impact on patients’ quality of life as compared to other oral mucosal diseases in dermatology patients has been shown elsewhere [13]. The present findings suggest that practitioners should notify type and number of OML and reported symptoms when making their treatment plan for this category of patients.

This study suggests that Sudanese patients with mucocutaneous diseases suffer moderate impairments of their OHRQoL, which is measureable by the Arabic version of the generic OIDP inventory. Moreover, eating, emotional problems and cleaning were the most frequently reported impacts, followed by problems with sleeping and speaking across subjects with and without OML. This compares to what has been observed among subjects with other medical conditions as well as with subjects from the general adult population in developed and developing countries [25,40,48]. The present frequency of OIDP ranging from 30 % to 44 % is comparable to the estimates of a national Greek survey (39 %), but is higher than those reported from national surveys in Norway (18 %) and Great Britain (12 %) [28,40]. On the other hand, this figure is lower than those observed in older adults in other cultures (50-60 %) [26], and from dental attendees in Khartoum (79 %) [31]. The present figures are also lower than those observed among Swedish adult patients (50-54 %) reporting regular medication according to the Anatomical Therapeutic Chemical classification system and having specific diagnoses of diseases categorized according to the WHO International Classification of Diseases, the ICID-10 [48].

Some limitations should be considered when interpreting the results. First, the cross sectional design restricts ability to make inferences with respect to the direction of the observed associations. Secondly, being a hospital based study; it is not possible to generalize findings to any larger population of mucocutaneous diseased individuals inside or outside Khartoum. Nevertheless, as KTH is the largest public main referral hospital in Sudan, receiving patients referred from all district in Sudan, the dermatology clinic-outpatients may capture the variety in characteristic of patients with skin diseases. In addition, self- reports and a recall period of 6 months can result in underestimation of health consequences, but might provide valid estimate for ultimate impact [49]. Self-selection and non-response bias might have influenced the results as patients were probably more likely to respond when they had OML. The present study suffered from lack of information regarding non-responders and thus non –response biases are difficult to estimate. Moreover, with respect to the diversity of the types of OML, the present figures might be biased towards those for which people are more inclined to seek treatment, whereas other conditions are less likely to be identified in hospital based prevalence studies. Absence of normative OIDP scores of the general Sudanese adult population, further limits possibility to use the general population as control group. Moreover, it should be acknowledged that the observations related to specific types of OML disease groups were based on small numbers and that the reported impacts cannot be attributed to specific diseases, symptoms and lesions. On the other hand, the generic OIDP scores might be compared across oral diseases and across specific patient groups and the general population. A generic OHRQoL instrument, such as the OIDP could help dermatologists to detect oral impacts, improve the patient doctor communication and provide the basis for better management of the dermatological patients, involving patients’ as well as the doctors’ perspectives.

## Conclusions

OIDP was more frequently affected among skin diseased patients with than without OML. The frequency of the impacts differed according to the number of type of OML, oral symptoms, and OML disease groups. Dentists and dermatologists should pay special attention to skin diseased patients because they are likely to experience oral impacts on daily performances.

## Competing interests

The authors declare that they have no competing interests.

## Authors’ contributions

NMS was the main author conceived and designed the study, collected data, performed statistical analysis and drafted the manuscript. ACJ was supervising and guiding the whole work and confirmed and approved all the diagnosis of oral lesions. RWA facilitated the field work. HS was the main dermatologist who examined and diagnosed all the patients. ANÅ was the main-supervisor of the present study, participated and guided the study design and has been actively involved in all stages throughout the work, especially statistical analyses and epidemiological analyses of data. All authors read and approved the final manuscript.

## Pre-publication history

The pre-publication history for this paper can be accessed here:

http://www.biomedcentral.com/1472-6831/12/19/prepub
